# Tailoring Hydrogel Structures: Investigating the Effects of Multistep Cellulose Defibrillation on Polyvinyl Alcohol Composites

**DOI:** 10.3390/gels10030212

**Published:** 2024-03-21

**Authors:** Gabriel Goetten de Lima, Bruno Bernardi Aggio, Alessandra Cristina Pedro, Tielidy A. de M. de Lima, Washington Luiz Esteves Magalhães

**Affiliations:** 1 Materials Research Institute, Technological University of the Shannon, N37HD68 Athlone, Ireland; 2 Graduate Program in Engineering and Materials Science, Federal University of Parana, Curitiba 12516-410, Brazil; 3 EMBRAPA Florestas, Colombo 83411-000, Brazilalecristinapedro@yahoo.com.br (A.C.P.); washington.magalhaes@embrapa.br (W.L.E.M.)

**Keywords:** PVA, nanofibrillated cellulose, composite hydrogels, freeze–thawing, kraft process

## Abstract

Defibrillating cellulose through various grinding steps and incorporating it into hydrogels introduces unique properties that warrant thorough exploration. This study investigates cellulose defibrillation at different steps (15–120) using an ultra-fine friction grinder, blended with high-molecular-weight polyvinyl alcohol (PVA), and crosslinked via freeze–thawing. A critical discovery is the influence of defibrillation on the hydrogel structure, as evidenced by reduced crystallinity, thermal degradation, and the enhanced swelling of PVA chains. Despite an increased elastic modulus of up to 120 steps, the synthesized material maintains remarkable strength under hydrated conditions, holding significant promise in biomaterial applications.

## 1. Introduction

From cellulose plant fibres, it is possible to obtain nanocellulose compounds that are shaped as fibrils and possess many advantages like renewability, biodegradability, nontoxicity, and a high surface area with many hydroxyl groups [1]. However, in order to obtain this nanostructured material, it is necessary to extract it from the plant cell wall. Mechanical grinding is the most attractive for being environmentally friendly and its ability to be performed on a large scale [2], presenting a good distribution of sizes of nanofibers compared to other treatments [3].

One of the many usages of nanocellulose is its potential filler aspect, which is used to improve mechanical properties, transparency, and many other characteristics of polymeric materials [4]. A recent application uses the hydrophilic nature of nanofibrillated cellulose as a reinforcement agent for hydrogels [5]. Hydrogels from polyvinyl alcohol (PVA), created using the freezing–thawing methodology, have been suggested in the literature, but they present poor elasticity and limited adhesion in a swollen state; therefore, alternatives of blending this polymer with cellulose have been proposed [6]. Nanocellulose as a filler presents many benefits. However, due to the strong intermolecular bonds between nanofibrils, they cannot be dissolved in common solvents but are easily dispersed. This leads to a strong viscosity (Supplementary Video S1), which depends on the concentration, dimension, and surface character, leading to an increased difficulty in processing and overall costs [7].

Reducing the concentration may be a feasible approach, since it is possible to achieve increased defibrillation of cellulose without impeding the continuous flow in processing machines. Achieving a higher yield of nanocellulose is also possible by increasing the number of grinding steps, a good approach if no pretreatments are possible or are nonviable for material synthesis.

Conventionally, physically crosslinked hydrogels from PVA synthesized with nanocellulose present many structural changes depending on the concentration and size of fibrils, as previously reported by our group [8]. The main backbone of a hydrogel structure changes based on the size of cellulose fibrils. Since it is not possible to perform more grinding steps on a highly concentrated nanocellulose suspension, this work investigates a more diluted regime (1 wt%) with more cycle steps, which this work evaluates from up to 7 h—corresponding to 120 steps. This is an interesting approach, since the current literature has included up to 2–4 h of grinding steps [9] and has used enzymatic pretreatment that can be performed for up to 24 h, which slightly improves fibre size [10,11]. Therefore, this study aims not only to understand the interaction mechanism of PVA toward an increasing number of nanocellulose fibrils, which may provide more site points for PVA chains to bind with cellulose, but also to obtain a good working range for the applicability of this material.

Nanocellulose ground at various steps and blended within PVA hydrogel can present unique features depending on the nanocellulose yield ratio, extracted by mechanical treatment. Such features can be exemplified by the mechanical properties, which increase in a dried and swollen state when increasing the nanocellulose ratio.

Therefore, this work investigates the effect of a low concentration of kraft pulp cellulose, 1 wt%; defibrillated at various steps, 15, 30, 60, and 120; blended with PVA; and crosslinked using freeze–thawing. Functionalization between nanocellulose and PVA occurred, modifying the morphology and thermal degradation of this material.

## 2. Results and Discussion

### 2.1. Nanosuspension Characteristics after Defibrillated at Multiple Steps

The SEM images of the nanosuspension (Supplementary Materials S1.i) exhibit a film with many pores in the first defibrillation step studied, i.e., step 15, but with increased defibrillation steps, the cellulose bundle becomes more homogeneous and denser. This is because the technique used to visualize the morphology of the nanosuspension utilizes a droplet from the nanosuspension within the sample holder, which then forms a film, and this film becomes denser with increasing defibrillation steps.

This effect can be further seen in the NIR technique (Supplementary Materials S1.ii) for the nanosuspension, in which increased absorbance values can be seen with increased defibrillation steps and the bands starting to become more defined for the nanosuspension. Therefore, the fibres are more homogeneous in size, and the suspension appears opaquer, leading to it absorbing more light. Finally, films produced using these gels, defibrillating at multiple steps, presented increased transmittance on UV–Vis (Supplementary Materials S1.iii) and are also related to their energy consumption in order to defibrillate the cellulose (Supplementary Materials S1.iv). However, they do not reach the high values of transmittance that are usually seen when pretreatments are performed, like enzymes (>70%) [12]. The work evaluated herein is focused on the effect of defibrillation on hydrogels and can provide good information on the interaction of low cellulose concentration as a reinforcement agent.

Overall, for 120 steps, defibrillation persists, which is based on the differences observed from the nanosuspension of 60 to 120. Since this method is a mechanical treatment, many factors are involved in the overall degree of defibrillation; however, previous works suggest using a degree of quality for the defibrillation using statistical analysis to use the nanosuspension commercial usage.

### 2.2. Morphology

**Figure 1:**
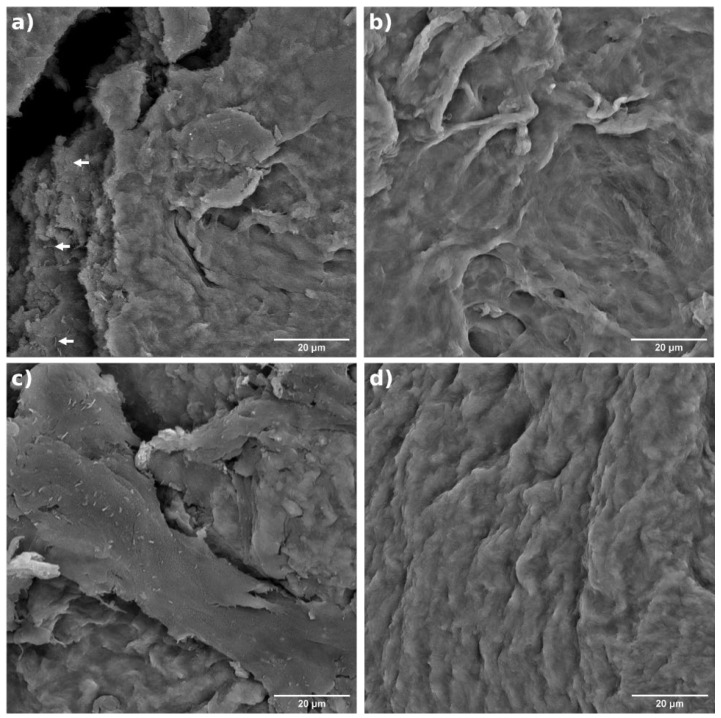
Surface morphology performed by SEM for PVA hydrogels samples containing cellulose defibrillated at different steps: (**a**) 15, (**b**) 30, (**c**) 60, and (**d**) 120.

**Figure 2:**
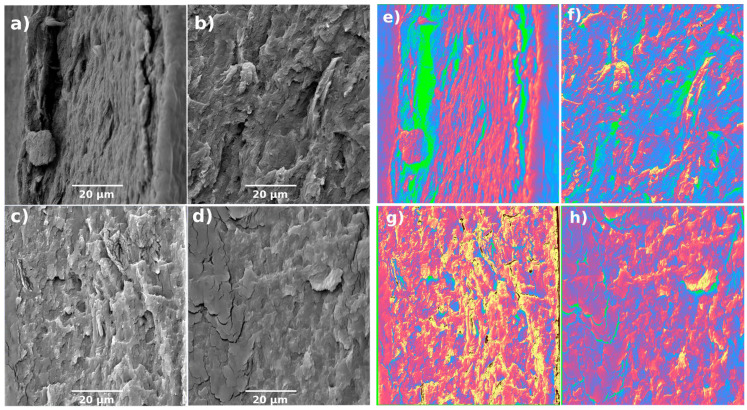
Cross-section (**a**–**d**) and processed colourmap (**e**–**h**) images for samples studied of PVA hydrogels with cellulose defibrillated at 15 (**a**,**e**), 30 (**b**,**f**), 60 (**c**,**g**), and 120 (**d**,**h**) steps.

The hydrogels samples exhibited a slight morphological difference depending on the number of cellulose defibrillation steps (Figure 1 and Figure 2). At low number of steps, the size of fibrils was qualitatively perceivable; for example, the arrows in Figure 1a (15 steps) indicate a fibre in the micro-range. As the number of steps increased, the fibre size decreased and became imperceptible (Figure 1b–d). Surface morphology also revealed a characteristic surface similar to a polymeric PVA material only at 120 steps [13], compared to the other samples. Additionally, there were differences in roughness among most of the samples.

The morphology suggests a relationship between surface roughness and both the cellulose defibrillation and the physical crosslink between PVA polymeric chains. It is possible that most of the well-defibrillated nanofibrils developed improved crosslinking within the polymer chains, resulting in the compacted structure observed at 120 steps.

These images illustrate the size-dependent binding kinetics between PVA and nanocellulose. Notably, the figure highlights a progressively rougher morphology with a decrease in the number of defibrillation steps. At 60 steps, faint fibres become discernible, while at 120 steps, their presence is less evident. The arrows in Figure 1 pinpoint an intriguing effect at 15 steps, capturing a moment when the film cracked, revealing the internal structure. This observation underscores the size-dependent characteristics of cellulose within the hydrogel matrix. The crack reveals a microscale structure, offering valuable visual evidence of the intricate relationship between defibrillation steps and the resulting hydrogel architecture.

The cross-sections of the samples (Figure 2) mirror the pattern observed on the surface, meaning that variations in the defibrillated steps impact the material’s structure. As the number of steps increases, the somewhat lamellar structure seen at the lowest milling step (Figure 2b), transforming into a more fragile aspect, typically associated with materials exhibiting increased resistance.

The cleavage planes performed for the cryofractures present a 3D morphology at low steps, transitioning to a more two-dimensional fracture plane with increasing steps. This trend is readily apparent from the colourmap filter implemented to these images (Figure 2e–h), where the greater variation in colour tones corresponds to increased roughness or topology variation. Therefore, the observed increase in roughness and the lamellar structure suggest a good interaction between PVA and nanocellulose. These findings align with previous work that employed ultra-fine friction grinding to obtain different samples from pure nanocellulose [14].

### 2.3. Microstructure

**Figure 3:**
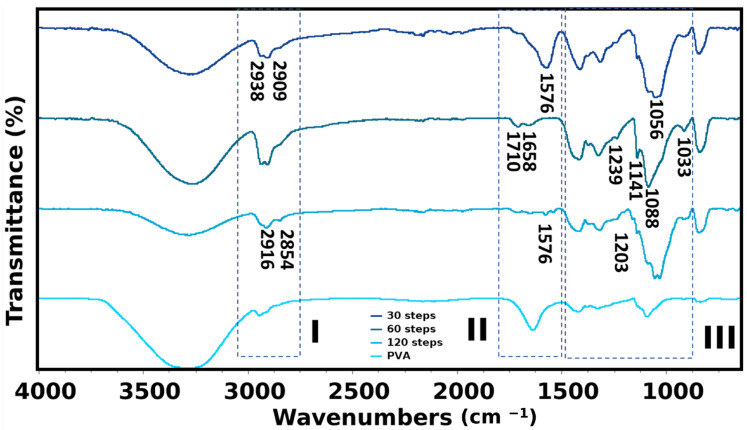
FTIR of PVA hydrogels and also containing cellulose defibrillated at different steps: 30, 60, and 120. Regions were assigned as to the most different profiles seen; the numbers on each assigned band mean that there is a perceived difference for that specific condition.

The infrared spectra of the hydrogels prepared at different steps present similar characteristics (Figure 3), consistent with previously reported bands for cellulose and PVA [8]. However, key differences emerge depending on the degree of cellulose defibrillation. Region I in Figure 3 highlights the -CH stretching region for PVA. The band at 2909 cm^−1^ (CH_2_ symmetric stretching [15]) is present in samples prepared with 30 and 60 defibrillations steps. At 120 steps, this band shift to 2916 cm^−1^, which aligns with a characteristic cellulose band (2916 cm^−1^ for CH_2_ stretching). In addition, the band at 2938 cm^−1^ (PVA CH_2_ antisymmetric stretching [15]) is observed for 30 and 60 defibrillation steps but appears weakly as a shoulder in the 120 step, potentially indicating intermolecular bonding. Conversely, the NFC cellulose band at 2854 cm^−1^ (CH stretching [15]) is faintly visible in defibrillated steps 30 and 60, but becomes more prominent at 120 steps. 

Nonetheless, even though differences were perceived, indicating that there is a transition between 60 steps and 120 during nanocellulose blending with PVA, this region represents various cellulose groups and is difficult to analyse in its entirely [16]. The FTIR spectrum for 15 steps is not shown due to difficulties in finding a homogeneous film, and 15 steps still contains clumps of cellulose bundles.

The next region (II—Figure 3) shows that the band at 1710 cm^−1^ of pure PVA (assigned to C=O stretching acetate group remaining from PVA) is present on all samples except for the one at 1658 cm^−1^ (PVA C-O stretching [17]). This band shifts, for the 30 and 120 steps, to 1576 cm^−1^. This shift can be related to contributions from both cellulose (antisymmetric stretching of carboxylic anion in hemicellulose [18] and PVA (C=C stretching vibration [19]). This observation suggests a potential interaction and functionalization between PVA and NFC, in agreement with previous works [20,21].

For the PVA region 1512–1285 cm^−1^ (III-Figure 3), the spectra are similar to all samples. Minor shifts are observed, likely due to the overlap with the main NFC band (1365–1319 cm^−1^, wagging vibration of cellulose CH_2_ [22,23]). This band is commonly used to calculate cellulose crystallinity. A higher shift in this band would indicate increased crystallinity; however, due to the presence of a similar band in PVA hydrogels, qualitative assessment of crystallinity from peak intensity is challenging.

Bands of PVA at 1261 cm^−1^ and 1239 cm^−1^ are seen only as a shoulder for 1261 cm^−1^, very weak, in the samples (assigned to -CO stretching vibration [8]). However, there is a preference for the band at 1239 cm^−1^, more defined. In addition, NFC band at 1203 cm^−1^ (C-O-C symmetric stretching) is seen as more defined with increasing defibrillated steps. Furthermore, the band at 1161 cm^−1^ of pure NFC (C-O-C asymmetric stretching vibrations associated with cellulose I and II [24]) is only present at 30 and 120 steps.

The band of PVA crystallinity 1141 cm^−1^ is less intense at 120 steps, and more defined at 60 steps, following the order 60 > 30 > 120. Also, the PVA band at 1088 cm^−1^ (C-O-C) is more defined in the 60 steps sample. However, the bands at 1056 cm^−1^ (1051 cm^−1^ on pure NFC) and 1033 cm^−1^ (1026 cm^−1^ on pure NFC) (characteristic bands of C–C, C–OH, C–H ring and side group vibrations [25]) are only seen for 30 and 120 steps.

Therefore, the spectra suggest that the interaction between the PVA and cellulose increases with increasing defibrillation steps, reaching a limit. When cellulose is not fully defibrillated, the PVA ordered chains are increased up to a certain point. Beyond this limit, celluloses are then the side chains that link with the PVA main backbone [8].

**Figure 4:**
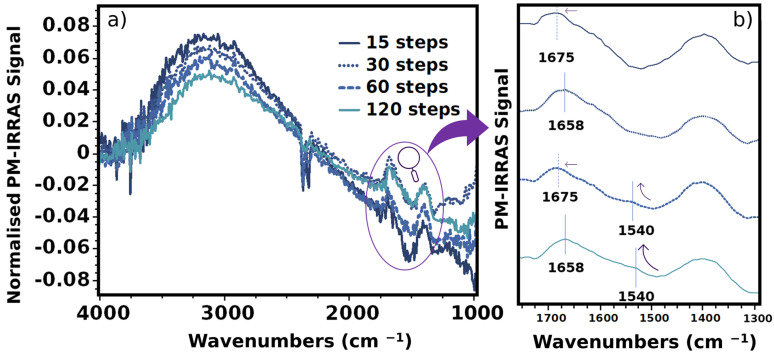
PM-IRRAS for the bulk signal of PVA hydrogels containing cellulose defibrillated at different steps, where (**a**) corresponds to the whole spectra region and purple circle indicates the region where a zoom with smoothing spectra was performed in (**b**).

To further verify this behaviour, PM-IRRAS was employed (Figure 4). While this is a powerful technique for comparing band intensities, this work only evaluated qualitatively because of the high noise produced in the film spectra. Nonetheless, a mathematical approach was used to estimate a profile solely from the bulk and the surface. This approach allows for some correlation with the IR spectrum [26] in order to perceive differences.

Consequently, the bulk spectra were given preference. Although the baseline exhibited some nonlinearity (which may be caused by the background material used), the OH-and CH-CH_2_ regions are seen as slopes. However, the most important bands were studied within the region of 1650–1300 cm^−1^, which is a region that presented the most distinct differences in the ATR-FTIR analysis.

Savitsky–Golay smoothing was used for this region, to perceive the highest-intensity absorbance value and detect any shift. Within this region, samples defibrillated with 15 and 60 steps had a slight blueshift compared with 30 and 120 step samples. This shift may correspond to a preference alignment towards PVA over NFC (Supplementary Materials S2).

The negative absorption band observed in the bulk region of these films suggests a preferential alignment of CH-O with water molecules at the surface level (for a detailed analysis of this discussion, please refer to the Supplementary Materials S3).

A small band is seen at 1540 cm^−1^ (Figure 4b), and its intensity increases with increasing defibrillation steps. This band is also seen in the FTIR spectra only for 120 steps sample (region II from Figure 3). It might be related to a double bond C=C, which appears relatively weak in pure PVA after physical crosslink is performed but is present in the pure NFC (Supplementary Materials S2). However, it increases with the addition of NFC and at higher defibrillation steps. This indicates that a strong interaction occurred within PVA and NFC with increasing defibrillation steps.

The spectroscopy techniques confirm that the unique band is seen for PVA and nanocellulose hydrogel at increasing defibrillation steps. This suggests that a possible functionalization process possibly occurred, since there is a higher yield of nanofibrils and significantly more interactions between PVA chains.

### 2.4. Mechanical Characterization

**Figure 5:**
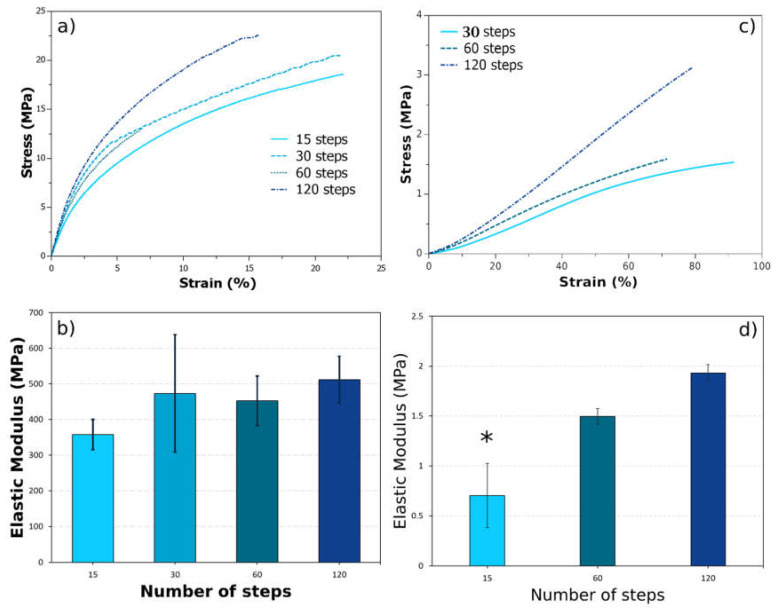
DMA tensile stress–strain mode for dried (**a**) and fully swollen (**c**) PVA+NFC hydrogels, containing cellulose defibrillated at different steps. (**b**,**d**) The elastic modulus calculated as the slope in stress–strain curves. (*) Statistically significant different by HSD Tukey test (*p* < 0.05).

Films consisting of NFC cellulose are known to be very strong [27]. Although the addition of PVA decreases the highest resistance in favour of a more hydrophilic character, the resulting hydrogel still presents a high elastic modulus, as shown previously [8]. For low cellulose concentrations, and increasing with defibrillation steps, it also shows variation on the elastic modulus (Figure 5) but remains weaker than that of hydrogels with higher nanocellulose concentration and is not statistically different. However, these hydrogels still demonstrate increased resistance and elastic modulus compared to pure F-T PVA (Supplementary Figure S4).

Increasing the defibrillation steps led to a rise in resistance. Notably, the films prepared with 60 steps exhibited exceptional behaviour, exhibiting a lower strain conversion with the same stress applied from all samples (Figure 5a,b). Nonetheless, their curve profile is similar to 120 steps, while 15 and 30 presented another unique profile; a characteristic similar to what has been suggested from the FTIR results.

When these films were swollen until they reached equilibrium, they were also analysed in tensile test mode under DMA, and due to the difficulty in measuring such slippery samples, only a part of the full force allotted from the equipment was used (Figure 5c,d). Measurements were only obtainable for samples with 30 or more defibrillation steps. Their profile also exhibited similar behaviour for the 60 and 120 steps samples, whereas the sample from 30 steps also exhibited a rather different profile. Interestingly, the only statistically significant difference in elastic modulus was observed for the sample with the least defibrillation. However, the elastic modulus values increased with the increasing defibrillation steps.

The elastic modulus values presented for these samples are quite high, if reflected on the low concentration of cellulose suspension (1%). Recent advancements in hydrogel development yielded tensile test elastic modulus values in the order of 6 MPa. These values are very attractive if defibrillated cellulose is to be considered as a blender for improvement in mechanical properties, as it has been shown for blends in polymeric materials [28,29]. The continued rise in resistance even after 120 defibrillation steps might be linked to the energy consumption profile (Supplementary Materials Figure S1.iv). This suggests that nanocellulose defibrillation was still ongoing at 120 steps, providing more anchor points for PVA to physically crosslink with nanocellulose.

### 2.5. Thermal Characterization

The first thermal properties analysed from these samples are the differential scanning calorimetry (DSC) to perceive differences around the crystalline PVA chains. The results indicate that the heat of fusion increased with increasing number of defibrillation steps (Supplementary Figure S4 and Table 1). However, at 120 steps, a decrease in the melting point area was observed, suggesting a reduction in PVA crystallinity. Nonetheless, both heat of fusion—related to the crystallinity—and melting point were lower compared to pure PVA. However, the melting point values are rather similar, and they increase for 120 steps.

The increase in crystallinity appears to be the dominant trend until the cellulose is defibrillated to 60 steps. After this point, the decrease may be related to overdefibrillated cellulose that may act as “anchor points” for cellulose chains, and hindering their self-association is no longer possible. During water evaporation, the nanocellulose fibrils within the system act as nucleation spots, and crystallization of PVA is induced by nucleation in the homogeneous solution with subsequent crystal growth. However, this process might be disrupted when in increased quantity.

**Figure 6:**
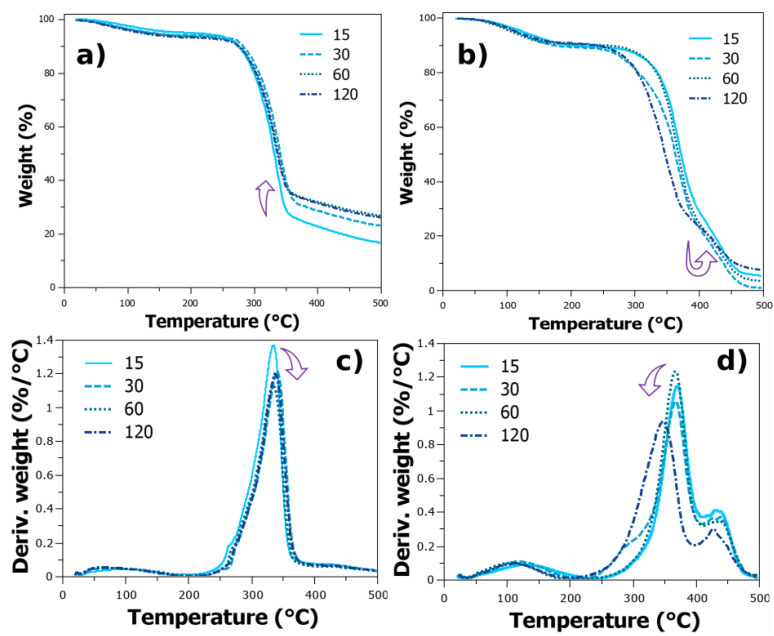
(**a**) Thermogravimetric analysis and its derivative for pure NFC films defibrillated at different steps (**a**,**c**) and PVA hydrogel samples blended with cellulose defibrillated at different steps (**b**,**d**). Arrows indicate the direction in which the main peak is shifting at increased defibrillation steps.

In terms of thermogravimetric analysis (TGA), the variation on defibrillation is also shown for pure NFC film (Figure 6). Increasing the defibrillation slightly increases the residues formation and their thermal resistance (arrow in Figure 6a,c and Table 1). It is possible that the increased compaction of fibres at higher defibrillation steps may raise the thermal gradient, providing increased residual charcoal. The thermal gradient rise is, as the simplest expression, a logarithm profile, and it may achieve a saturation which could be related to the number of the compacted fibres seen by the thermogravimetric analysis graph. The differential thermogravimetric analysis presents only one degradation stage, which corresponds to various process for cellulose degradation of glycosyl units and oxidation breakdown [8].

Table 1 encapsulates crucial thermal analysis data, combining TGA and DSC values for a comprehensive understanding of the hydrogel’s thermal behaviour. The TGA section outlines the maximum temperature of decomposition for the PVA+NFC hydrogel and the temperature at which 10% of its weight is decomposed. On the other hand, the DSC data delve into the melting point of PVA after varying defibrillation steps, along with corresponding heat flow values.

For the PVA with defibrillated cellulose hydrogels, a different profile is seen (Figure 6b,d). In this case, increasing the defibrillation steps seems to exhibit a decrease in thermal resistance (Table 1). Interestingly, the sample with cellulose defibrillated at 60 steps exhibited a profile similar to that of the 15 steps sample, mirroring the trends observed in the IR data regarding peak shifts and intensities. However, the sample with 30 defibrillation steps presented almost no residue. After this point, increased residues are seen (Figure 6b). It is important to remember that PVA was physically crosslinked after defibrillation; thus, the same profile as pure NFC was expected. However, it is possible that the interaction of PVA and the crosslinking agent altered the structural configuration, potentially relaxing these chains up to a point, related to the 60 steps, at which point residues begin to appear.

PVA is reported to have five main degradation stages, whereas the main ones are at 365 °C and 427 °C (Supplementary Figure S4c). The first is characteristic of oxygen functional groups’ removal and formation of polyene intermediate, while the last one is associated with chain breakdown yielding char and other small molecules. The thermal degradation behaviour slightly decreases after defibrillation steps, but the chain breakdown stage increases until 60 steps, whereas after 120 steps, it heavily decreases (Table 1 arrow in Figure 6d).

Samples of weakly defibrillated NFC+PVA hydrogels may be strictly associated with the cellulose degradation, mainly because of intermolecular bonds, and so the freeze–thaw weakly disrupts these forces. However, for more severely defibrillated cellulose, the thermal profile is more associated with the degradation of PVA, and few defibrillation steps follow the cellulose degradation pattern. A key feature is the increased degradation step of the main stage, up to 360 °C compared to 335 °C of pure NFC (Table 1).

### 2.6. Swelling Kinetics

**Figure 7:**
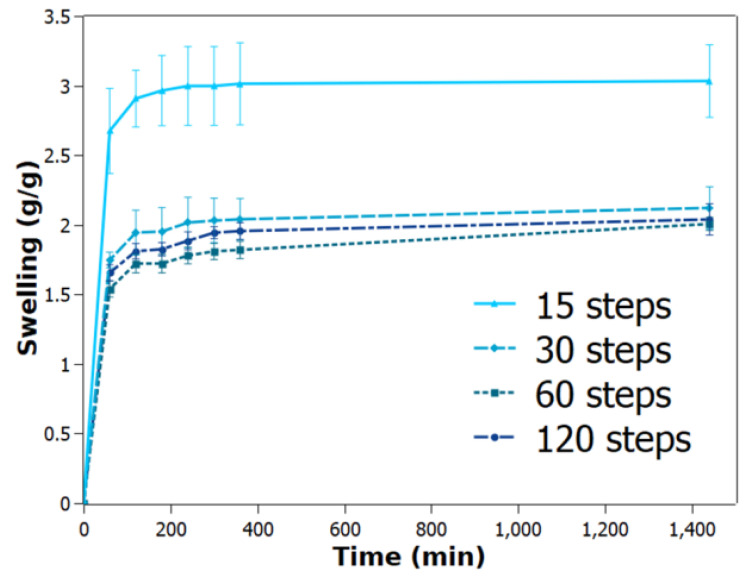
Swelling ratio of the studied PVA hydrogels containing defibrillated cellulose at different steps.

These hydrogels were allowed to swell in distilled water, and their weights were measured at specific time intervals. The swelling data were plotted against time (Figure 7). The results show that after 15 steps for the defibrillated cellulose, their swelling ratio significantly decreases, indicating a good crosslink between the PVA and cellulose. However, after 30 steps, these values start to decrease slowly, with similar values of swelling without significant differences.

Complementing the thermal analysis, Table 1 also incorporates parameters derived from the Schott equation model, offering essential insights into the hydrogel’s swelling characteristics. The constants obtained from this model play a pivotal role in elucidating the limits of water absorption by the hydrogel, providing a valuable reference for its swelling behaviour and its adherence to a well-established model.

Therefore, these hydrogels follow the Schott equation model, which describes a swelling process resulting from the polymer macromolecules unravelling after diffusion into solvent upon hydration. These samples correlate well with the model and predict the swelling rate quite well for all hydrogels, in which there is an increase after reaching 120 steps. The swelling rate decreases until 60 steps of defibrillating the cellulose, and decreases further from the pure PVA hydrogel (Supplementary Figure S4d), which presents a rather fast swelling rate. This suggests that the nanocellulose is able to control the rate of water absorption since the intertwined fibres within the structure compel for a more difficult penetration of water. However, these values after 15 steps were not statistically significant different and may well just follow the trend of 60 steps, i.e., approaching a constant value.

**Figure 8:**
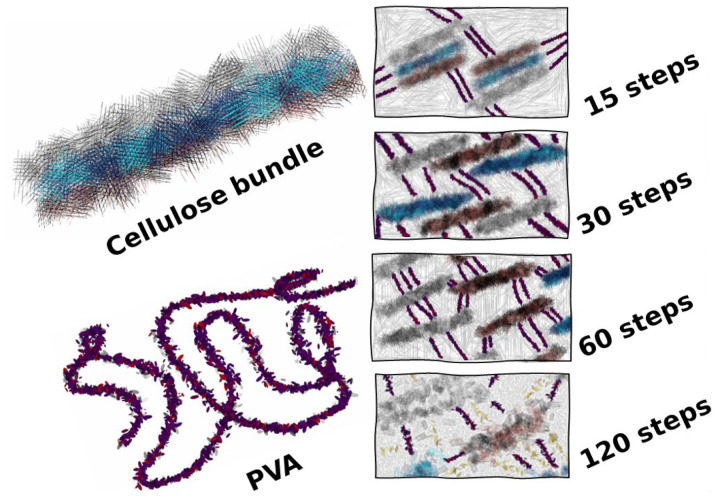
Diagram to illustrate the hypothesis of this work in which cellulose bundles are defibrillated by the mechanical forces; depending on the degree of defibrillation, the interaction between PVA by physical crosslinking changes. A saturation fn the interaction occurs at 60 steps, and at 120 steps, PVA is aggregated around the overdefibrillated fibres.

Since overdefibrillated samples might be well compacted, one would expect that this would present an impediment for the water to diffuse inside the hydrogel. This may indicate that overdefibrillated fibrils may present more functionalized groups that readily form interactions with water molecules upon water penetration. These attributes may also explain the maximum water uptake seen for 120 steps. In order to further enhance this hypothesis, a diagram was drawn to illustrate the effect of shear forces as correctly identified by a previous work [30]. These shear forces slowly defibrillate cellulose until the aggregates begin to disassemble and are directly related to the number of defibrillation steps. As 120 steps are reached, the interaction between PVA and NFC may change, as identified by our previous work [8] and by [31] (Figure 8).

Our work holds significance in several aspects. Firstly, it contributes to the understanding of the complex interplay between cellulose defibrillation and the properties of resulting hydrogels. This knowledge is crucial for tailoring cellulose-based materials with desired characteristics, thereby expanding their practical applications. Additionally, the optimized defibrillation steps identified in our study offer insights into the efficient utilization of resources, potentially influencing the economic feasibility of large-scale production. The enhanced properties of the PVA+NFC hydrogel open up possibilities for novel applications in the biomaterials field, showcasing the importance of this work in advancing sustainable and cost-effective material science.

## 3. Conclusions

This study unveils a critical relationship between the degree of cellulose defibrillation and the properties of PVA hydrogels. We observed a trade-off, where increased defibrillation from 15 to 120 steps significantly enhanced the mechanical properties of the hydrogels. The elastic modulus of dried films increased by 43% (from 350 MPa to 500 MPa), while the gel state modulus saw a nearly threefold increase (from 0.7 MPa to 2 MPa). However, this improvement came at the cost of decreased thermal stability, as evidenced by a reduction in PVA crystallinity. The melting point and heat of fusion of PVA decreased, with the heat of fusion dropping from 73 J/g to 22 J/g. Furthermore, FTIR and PM-IRRAS data suggest the formation of new interactions within the matrix, potentially involving C=C bonds. By establishing this interplay between defibrillation and hydrogel properties, our research provides valuable insights for optimizing the design of PVA hydrogels. This paves the way for the development of novel hydrogels with a desired balance of mechanical strength, thermal stability, and other properties for diverse applications in biomedicine and material science.

## 4. Materials and Methods

### 4.1. Materials

Materials used in this study were Mowiol^®^ 56–98, M.W. 195 kDa with 98.0–98.8 mol% hydrolysis (Sigma-Aldrich, São Paulo, Brazil) and bleached eucalyptus kraft pulp (Suzano Papel e Celulose, São Paulo, Brazil) for the raw material of nanofibrillated cellulose (NFC) preparation.

### 4.2. Preprocess of Cellulose Suspension

The kraft pulp was preprocessed using a 450 W blender for 10 min, using a concentration of 1 wt% with distilled water as solvent.

### 4.3. NFC Cellulose Suspension

Cellulose suspension was defibrillated using the Super Masscolloider (Masuko Sangyo Co. Ltd., Kawaguchi, Japan). The technical parameters related to the ultra-fine friction grinder in obtaining the cellulose nanofibrils were rotation: 1500 rpm and distance between discs: 0.1 mm. Moreover, at various steps, part of the gel was retained in order to blend with PVA—15 steps, 30 steps, 60 steps, and 120 steps. Two sets of gels were obtained, one containing pure NFC for thermal studies, and another for blending with PVA. To avoid any differences between the gels, the stone disks and the apparatus were not removed, and cleaning was performed by flowing water through the disks as a step method until no cellulose was seen from the flowing water. Pure gel was analysed by bench NIR 900 (FEMTO, São Paulo, Brazil). The spectra were collected between wavelengths of 1100 nm and 2500 nm, with a resolution of 2 nm using diffuse reflection mode. A droplet of the gel was used for the SEM analysis (TESCAN Performance in Nanospace, Brno, Czech Republic). The films, casted in Petri dishes (20 mL), were analysed with UV–Vis performed in transmittance mode (UV–Vis model 1800 (Shimadzu Corp, Kyoto, Japan)).

### 4.4. Hydrogel Synthesis of NFC Cellulose with Polyvinyl Alcohol

Polyvinyl alcohol was dissolved using the same concentration (5% *w/v*) for all cellulose suspensions at 80 °C with constant stirring until complete solubilization of PVA was achieved (~2 h). Afterwards, samples were gently poured into ice cubes using a constant weight of solution for all conditions (~25 g). Finally, the samples were rapidly frozen to a constant temperature of −70 °C for two hours using an ultra-freezer. The frozen solutions were then thawed in a controlled temperature environment at 25 °C; this freeze–thawing procedure was performed six times, and after completion of the physical crosslink, samples were dried in an oven at 30 °C.

### 4.5. Attenuated Total Reflectance-Fourier Transform Infrared Spectroscopy (ATR-FTIR)

The FT-IR spectra were obtained from a spectrometer (Perkin Elmer, São Paulo, Brazil), model FT-IR/NIR Frontier, coupled with an attenuated total reflectance (ATR) accessory with zinc selenide (ZnSe) crystal surface. A resolution of 4 cm^−1^ with arithmetic average of 64 scans was used in the wavenumber range of 4000–550 cm^−1^.

### 4.6. Polarization Modulation Infrared Reflection Absorption Spectroscopy (PM-IRRAS)

PM-IRRAS experiments were carried out using a KSV-Nima equipped with polarization modulation unit (ZnSe modulator PEM-100, Hinds Instruments, Hillsboro, USA) and MCT detector. The measurement range expressed in the wavenumber was between 800–4000 cm^−1^, spectral resolution was 8 cm^−1^, and the incident angle was 79°. The equipment operated at the 50 kHz frequency, while the frequency of the highest amplification was set to 1500 cm^−1^ and the retardation was 0.5. Each measurement consisted of 1200 scans; thus, the measurement time was 2 min. Scans of the background, standard glass-slide, and samples were taken individually.

In order to obtain data from the surface and the bulk material, data processing was performed according to [32]. The surface reflectivity factor and bulk reflectivity factor were calculated according to Formulas (1) and (2), respectively.

(1) $$\mathrm{Surface}\;\mathrm{reflectivity}\;\mathrm{factor}\;=\left(\frac{Sample\;R_{dif}}{Reference\;R_{dif}}\right)-1$$

(2) $$\mathrm{Bulk}\;\mathrm{reflectivity}\;\mathrm{factor}\;=\left(\frac{Sample\;R_{avg}}{Reference\;R_{avg}}\right)-1$$

where *R_dif_* and *R_avg_* are the collected difference, R_s_ − R_p_, and sum, R_s_ + R_p_, intensities of the p- and s-polarized light, respectively. For analysis of the surface, the spectra were normalized, while for bulk, the spectra were also smoothed with a second-order Savitsky–Golay function using 10 interval setpoints in order to perceive key differences within peaks selected at the specific region of 1650–1300 cm^−1^.

### 4.7. Scanning Electron Microscopy

The hydrogel morphology was analysed using the scanning electron microscopy (TESCAN Performance in Nanospace, Brno, Czech Republic) with the backscattered electron (BSE) mode. Prior to imaging, the samples were sliced to obtain cross-sectional regions and were also gold-sputtered for coating using Baltec SCD 005 for 110 s at 0.1 mBar vacuum. Images were recorded at an accelerating voltage 20 kV. For cross-section images, samples were cryofractured using liquid nitrogen and the images were processed in order to obtain a colourmap, using the colorize (with Colormap) function filter from G’MIC within GIMP software [33]. The interpolation used was the Lanczos with five tones.

### 4.8. Thermodynamic Analysis

For differential scanning calorimetry (DSC), samples (weight of 9–12 mg) were encapsulated in sealed aluminium sample pans. A temperature ramp from 0 °C to 250 °C with 10 °C/min rate was used, with an empty crimped pan as a reference.

Thermogravimetric analysis (TGA) curves were obtained with a heating rate of 10 °C/min until 600 °C using alumina pans with samples weighing around 5.0 mg. The experiments were carried out under nitrogen flow of 50 mL min^−1^, in Q600 TA Instruments equipment.

Dynamic mechanical analyses, DMA, were performed on DMA Q800 TA Instruments equipment using the film tension clamp. Stress–strain tests were performed in dried and fully swollen hydrogels with a ramp force of 1 N/min up to 18 N, and the elastic modulus was calculated from the initial linear region—0.002% strain of the stress–strain curve. Sizes for samples used for DMA were 10 × 12 × 0.1 mm^3^. All tests from DMA were performed using three scans per sample. Hydrated samples were first allowed to swell for up to ten days, a period confirmed to be in the maximum swelling ratio; afterwards, they were measured using the same film tension clamp and manually sprayed with water at interval times in order to not lose the maximum swelling state while under test.

### 4.9. Kinetics of Swelling of Hydrogels

For the swelling measurement, PVA hydrogels were measured gravimetrically. To measure the swelling kinetics, the preweighed samples were immersed in deionized water. Periodically, samples were taken, the excess surface solution was gently removed with a paper towel, and the swollen samples were weighed at various time intervals. The swelling ratio percentage of a hydrogel was calculated and plotted against time.

In addition to the swelling analysis, the swelling data were obtained using a kinetic model of the second-order equation proposed by Schott [34]:

(3) $$\frac{t}{W}=A+\mathrm{Bt}$$

where B = W_∞_^−1^ and A = (dW/dt)^−1^; W and W_∞_ are the water absorption capacity at time t and at equilibrium, respectively; B is the inverse of the theoretical equilibrium swelling; and A is the inverse of the initial swelling rate. By plotting t/W against t, it is possible to obtain, from the angular and linear coefficient, the values of W_∞_ and K_s_, where K_s_ is the swelling rate constant (g/g min) and is related by K_s_ = (A × W_∞_^2^)^−1^.

### 4.10. Statistical Analysis

For parametric data, post hoc Tukey-HSD test was performed using the “agricolae” R package v.1.3-7.

## Figures and Tables

**Table 1:** Thermal analysis and theoretical adjustment values using Schott equation for the studied hydrogels.

Samples	TGA			DSC—PVA		Schott Equation Model			
	T_max_ (°C)		T_10_ (°C)	T_m_ (°C)	ΔH_f_ (J/g)	R^2^	K_s_	W_∞_ Calc	W_∞_ Obs
	1st Stage	2nd Stage							
PVA	365 ± 3	427 ± 4	268 ± 3	226 ± 2	73 ± 6	0.9999	0.014	3.48	3.46
15	369 ± 5	435 ± 3	204 ± 2	223 ± 2	24 ± 3	0.9999	0.077	3.04	3.03
30	367 ± 5	436 ± 4	173 ± 2	222 ± 2	26 ± 2	0.9998	0.038	2.13	2.12
60	366 ± 4	440 ± 5	256 ± 3	223 ± 2	27 ± 3	0.9988	0.019	2.03	2.00
120	347 ± 4	426 ± 4	238 ± 1	223 ± 2	22 ± 3	0.9998	0.031	2.05	2.03

For DSC—T_m_: melting point of PVA; ΔH_f_: heat of fusion for PVA hydrogels; TGA—T_max_ maximum temperature of decomposition for two stages seen on the thermo curves and T_10_ temperature to decompose 10% of the hydrogel. K_s_: swelling rate, W_∞_ calc: theoretical, and W_∞_ obs experimental value of equilibrium swelling.

## Data Availability

Data will be made available upon request.

## Supplementary Materials

The following supporting information can be downloaded at: https://www.mdpi.com/article/10.3390/gels10030212/s1. Supplementary Video S1; Figure S1.i: SEM morphology of pure defibrillated cellulose at (a) 15 steps, (b) 30 steps, (c) 60 steps, and (d) 120 steps. Figure S1.ii: NIR spectra of the pure cellulose gels defibrillated at various steps. Figure S1.iii: UV–Vis of the pure cellulose films defibrillated at various steps. Figure S1.iv: Energy consumption of pure cellulose (1 wt%) using water as solvent in ultra-friction grinding. Figure S2: FTIR of pure (PVA) powder and after physical crosslinking (PVA F/T), and also the spectra of pure MFC. Assigned bands and highest intensity values were added in the figure for clarity. Text S3: PM-IRRAS surface signal. Figure S3: PM-IRRAS for the surface signal. Figure S4. Pure PVA F-T (5 wt/vol.%) data for DMA tensile tests (a) with its elastic modulus, (b) DSC data with its melting point and heat of fusion values; (c) TGA with main degradation events; (d) swelling with model theoretical values using Schott equation. Figure S5: Differential scanning calorimetry profile for the studied hydrogels, the zoomed region exhibits the melting point of PVA. References [35,36] are cited in the Supplementary Materials.
