# Supplementary material for: Tailoring Hydrogel Structures: Investigating the Effects of Multistep Cellulose Defibrillation on Polyvinyl Alcohol Composites
Source: Gels. 2024 Mar 21;10(3):212. doi: 10.3390/gels10030212 (PMC10970658; doi:10.3390/gels10030212)
Supplement: Supplementary file 1 [file gels-10-00212-s001.zip › Supplementary material.pdf]

S.1 Pure defibrillated cellulose

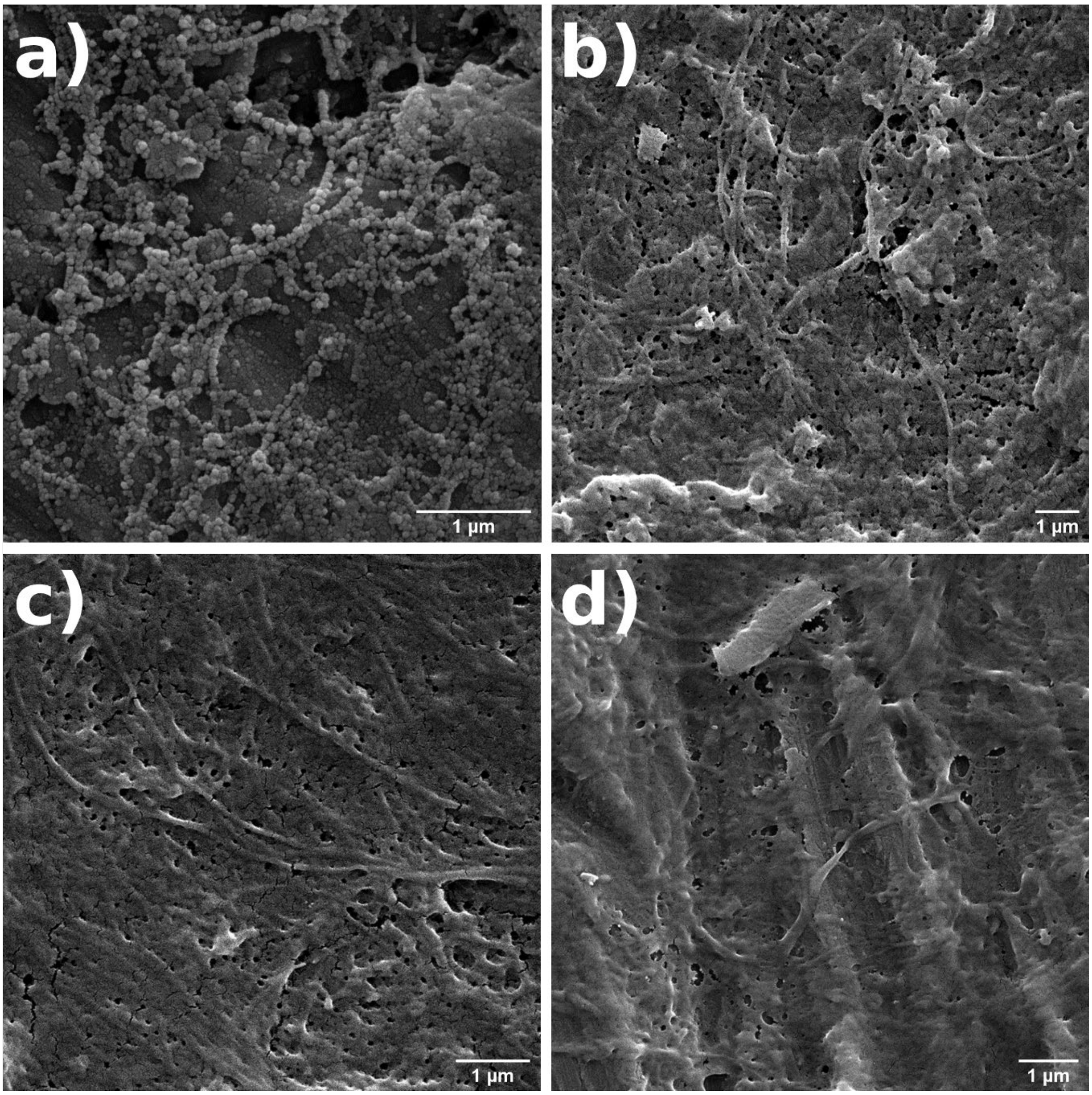

Figure S.1.i. SEM morphology of pure defibrillated cellulose at a) 15 steps, b) 30 steps, c) 60 steps and d) 120 steps.

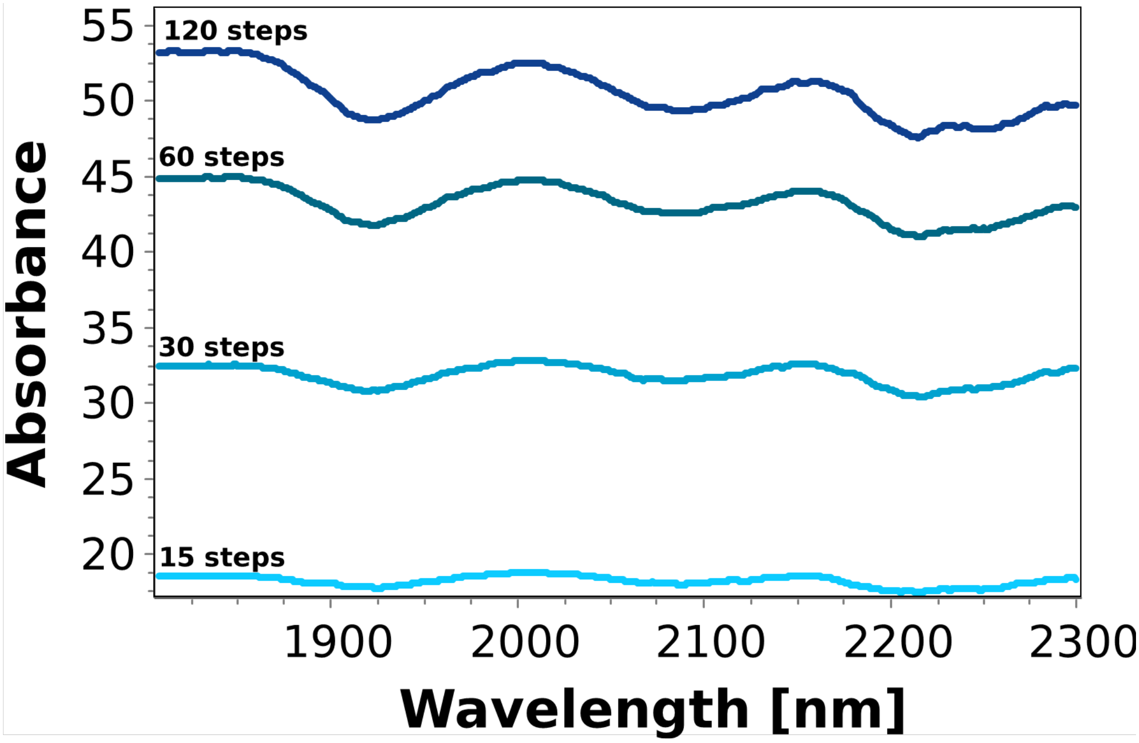

Figure S.1.ii. NIR spectra of the pure cellulose gels defibrillated at various steps.

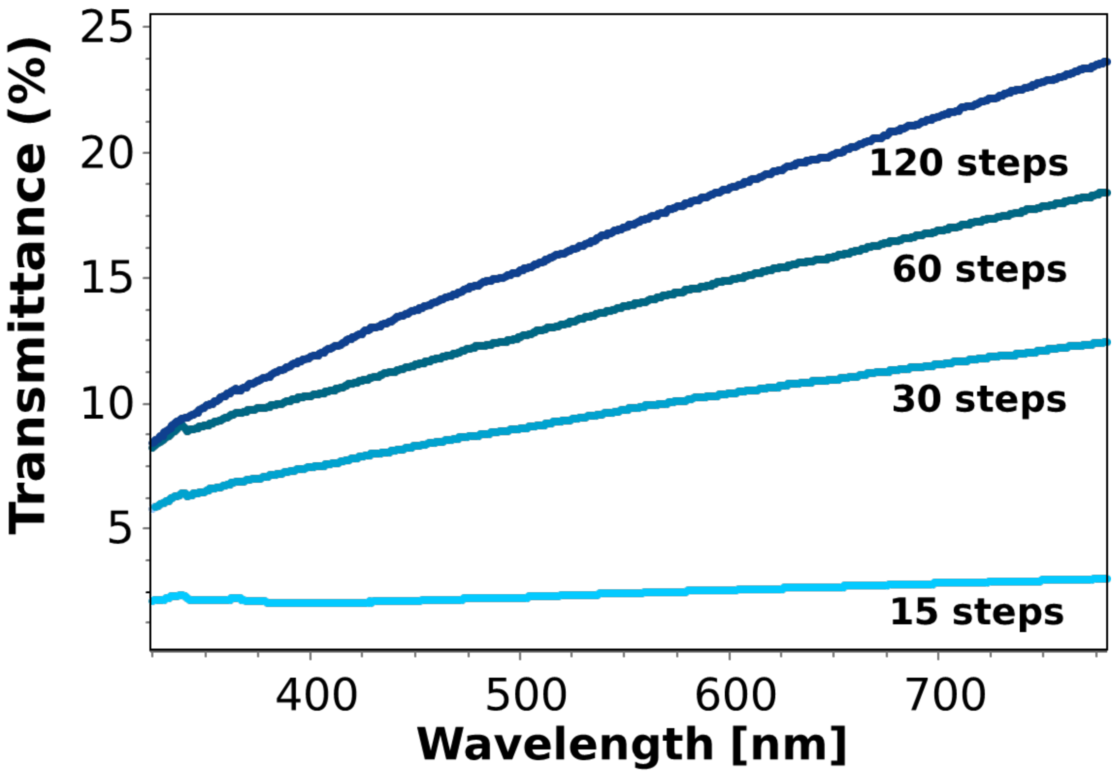

Figure S.1.iii. UV-VIS of the pure cellulose films defibrillated at various steps.

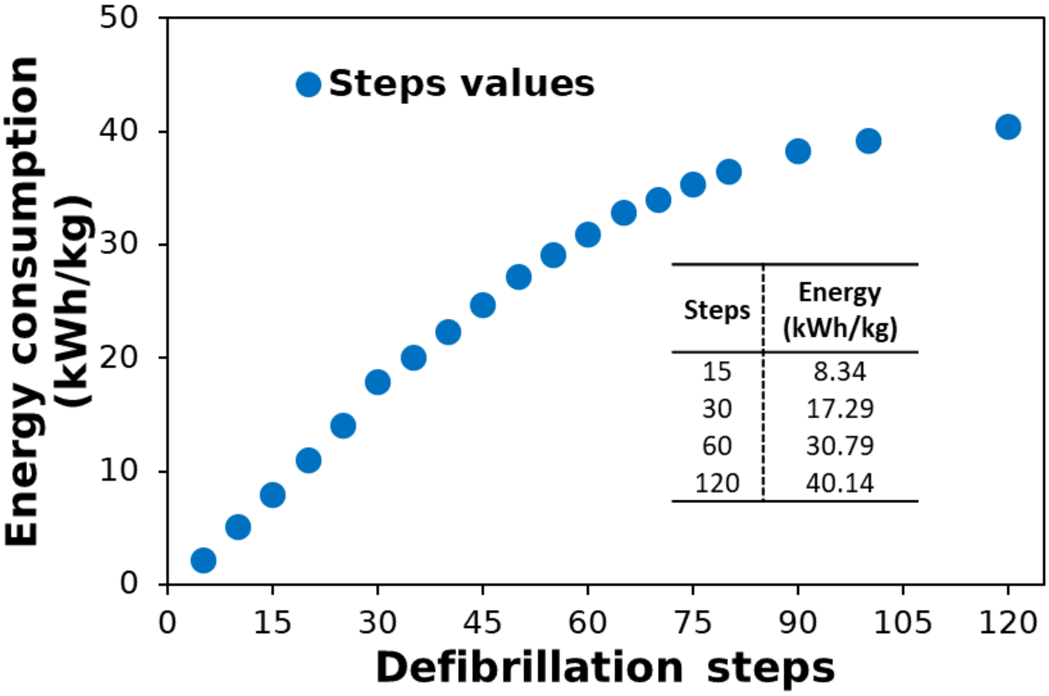

Figure S.1.iv. Energy consumption of pure cellulose (1 wt%) using water as solvent in ultra-friction grinding.

S.2 Spectra of pure PVA and MFC

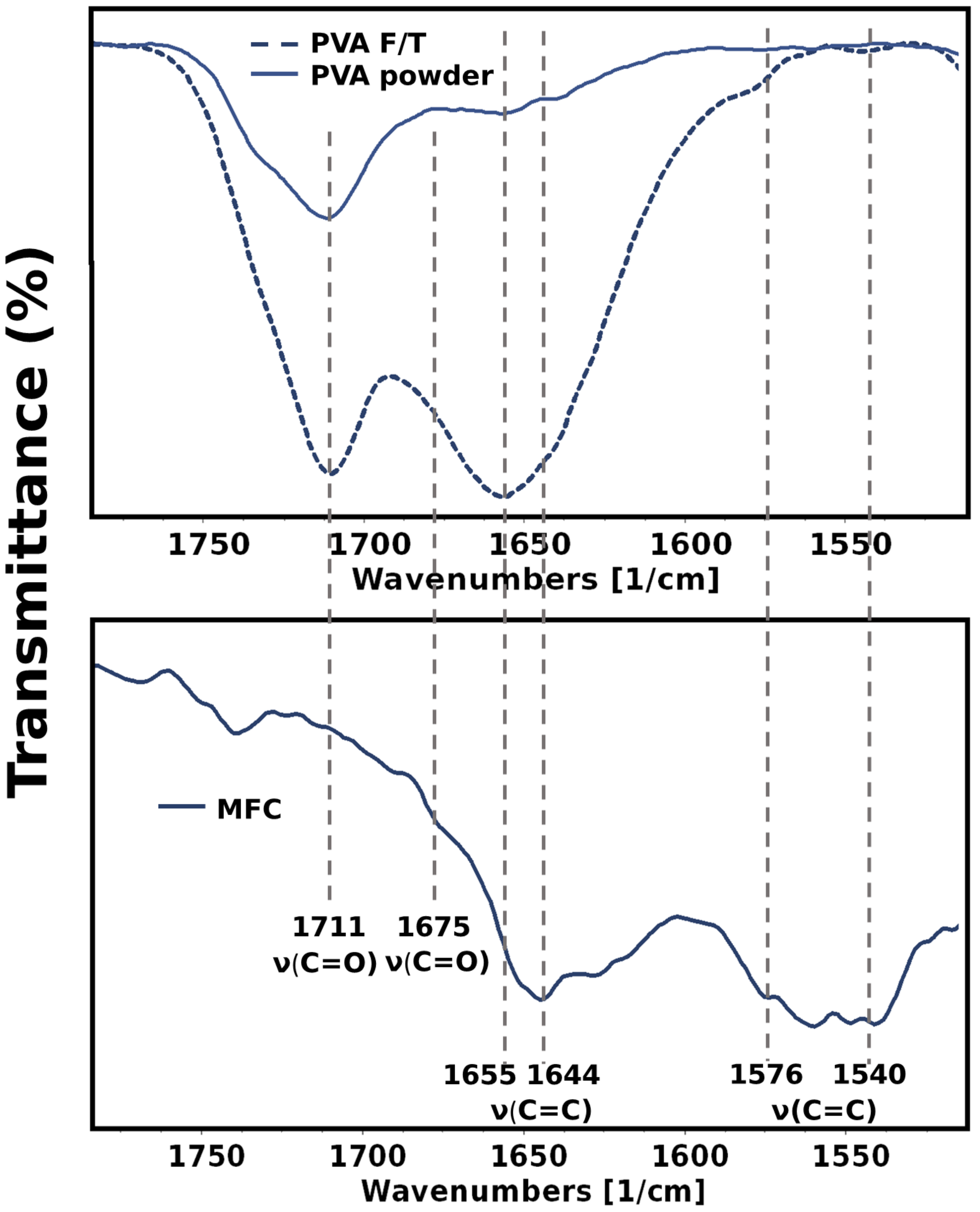

Figure S.2. FTIR of pure (PVA) powder and after physical crosslinking (PVA F/T) also the spectra of pure MFC. Assigned bands and highest intensity values were added in the figure for clarity.

S.3 PM-IRRAS Surface Signal

The primary attribute seen on the surface aspect of IRRAS presents a main band attributed to CH-O. In general, cellulose chains forms flat sheets that are interconnected by OH-O hydrogen bonds (Chami Khazraji & Robert, 2013b). These sheets are stacked by van der Waals dispersion forces and weak CH-O hydrogen bonds. The surface is considered as a

defect in materials science, and therefore, this defect presents differences to the bulk material whereas half of the cellulose intermolecular hydrogen bonding is lost but is compensated by hydrogen bonds with water molecules (Chami Khazraji & Robert, 2013a). This is because cellulose chains are very reactive with water molecules and form hydrogen bonds as soon as the cellulose chains come in contact with water molecules and is the reason the main band is seen at Figure A.3.

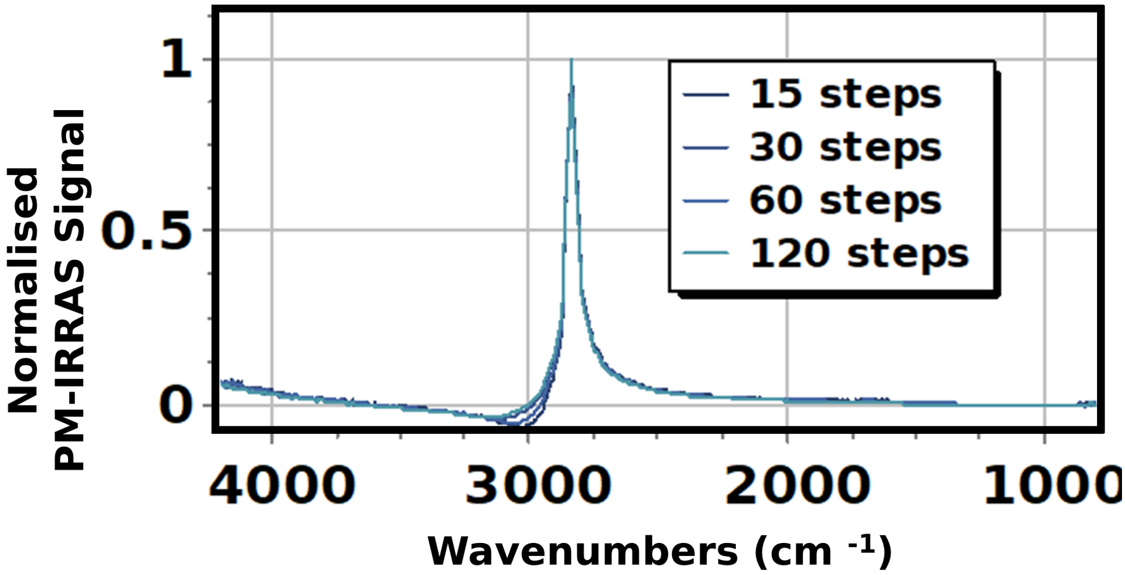

Figure S.3. PM-IRRAS for the surface signal.

References.

Chami Khazraji, A., & Robert, S. (2013a). Interaction Effects between Cellulose and Water in Nanocrystalline and Amorphous Regions: A Novel Approach Using Molecular Modeling. *Journal of Nanomaterials*, *2013*, 1–10. https://doi.org/10.1155/2013/409676

Chami Khazraji, A., & Robert, S. (2013b). Self-Assembly and Intermolecular Forces When Cellulose and Water Interact Using Molecular Modeling. *Journal of Nanomaterials*, *2013*, 1–12. https://doi.org/10.1155/2013/745979

S.4 Pure PVA hydrogel (F-T)

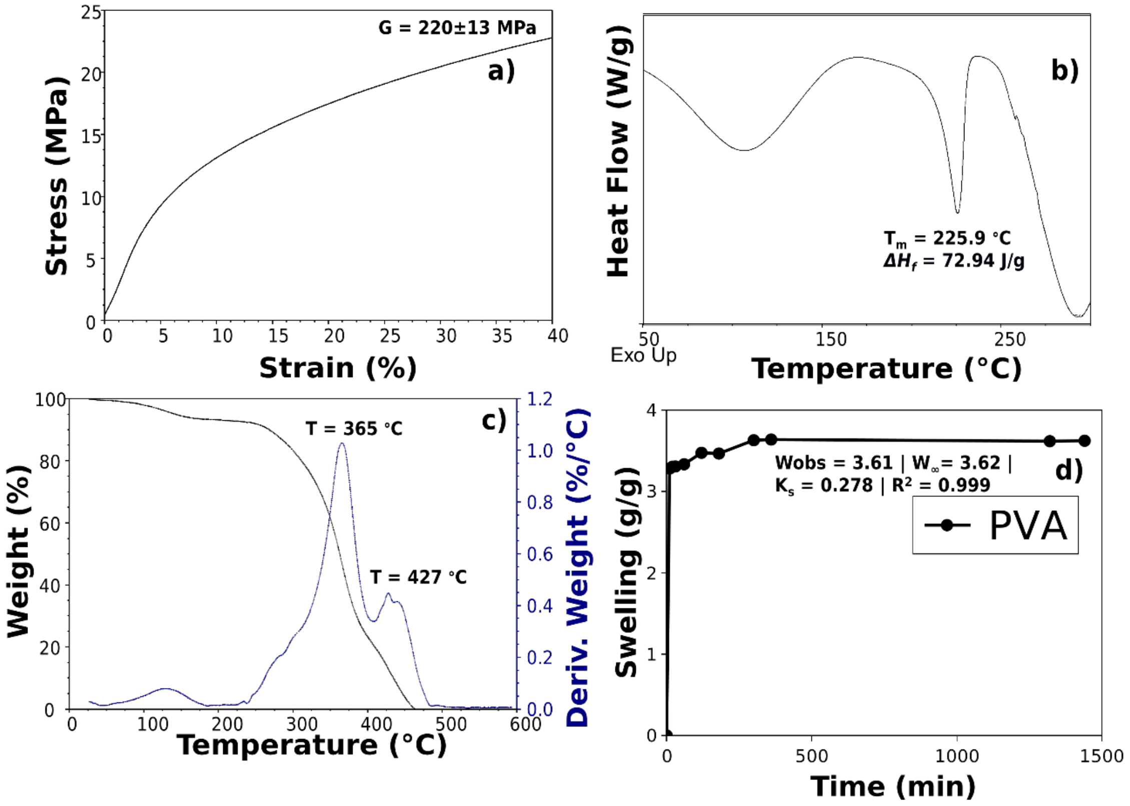

Figure S.4. Pure PVA F-T (5 wt/vol.%) data for DMA tensile tests (a) with its elastic modulus, (b) DSC data with its melting point and heat of fusion values; (c) TGA with main degradation events; (d) swelling with model theoretical values using Schott equation.

S.5 DSC results graph

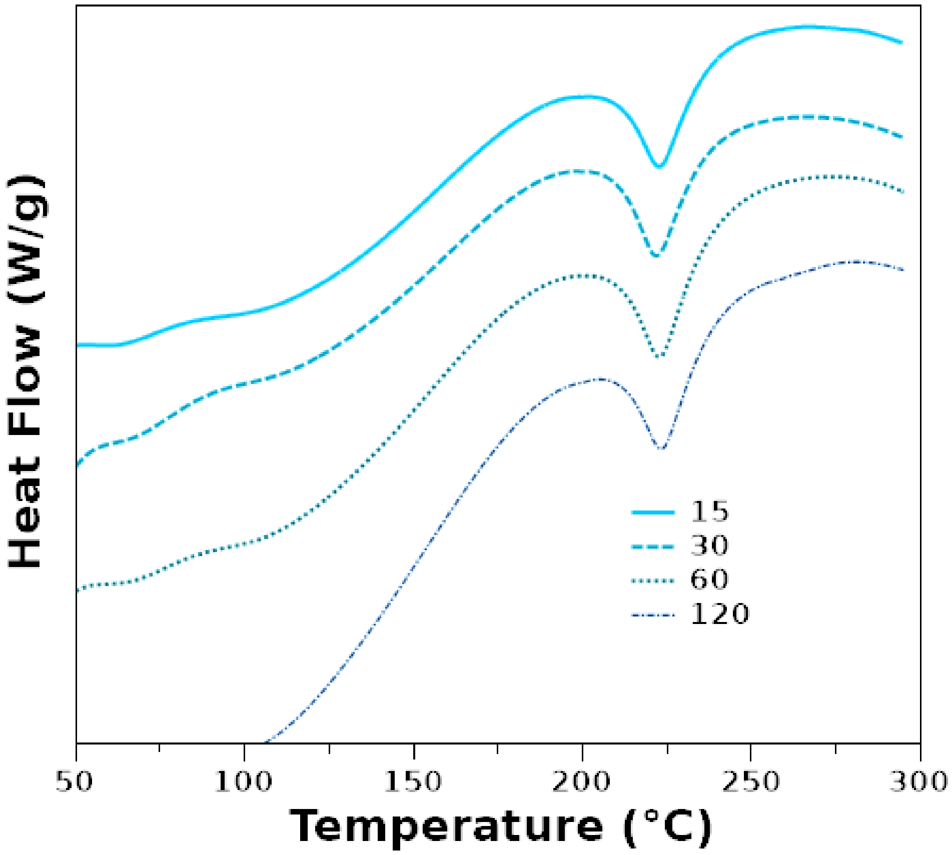

Figure S.5.a. Differential scanning calorimetry profile for the studied hydrogels, the zoomed region exhibits the melting point of PVA.
